# Evolution of contact and alarm calls in the Kenyan endemic Hinde’s babbler (Aves: Passeriformes)

**DOI:** 10.1186/s12862-018-1222-1

**Published:** 2018-07-17

**Authors:** Jan Christian Habel, Martin Husemann, Werner Ulrich

**Affiliations:** 1 Terrestrial Ecology Research Group, Department of Ecology and Ecosystem Management, Technische Universität München, Hans-Carl-von-Carlowitz-Platz 2, D-85354 Freising, Germany; 2 Centrum für Naturkunde, Universität Hamburg, D-20146 Hamburg, Germany; 3 Department of Ecology and Biogeography, Nicolaus Copernicus University in Toruń, Pl-87-100 Toruń, Poland

**Keywords:** Altitude, Geographic isolation, Environmental conditions, Social structures, Birds, Cooperative breeder, Bioacoustics

## Abstract

**Background:**

Spatial isolation, diverging environmental conditions and social structures may lead to the differentiation of various traits, e.g. molecules, morphology and behaviour. Bird calls may provide important information on effects of geographic isolation and may reflect diverging ecological conditions related to altitude. Furthermore, bird calls are strongly shaped by the social behaviour of species. The Kenyan endemic bird Hinde’s Babbler, *Turdoides hindei*, is a cooperative breeder existing in distinct family groups. The species occurs in five isolated population groups at different altitudes across its distribution range in south-eastern Kenya. With this model species we test for potential effects of geographic isolation, diverging altitudes, and social structures. We recorded and analysed contact and alarm calls of *T. hindei*, including its entire distribution range and all existing population groups.

**Results:**

Our data show significant differentiation of call characteristics among population groups across the species’ distribution range. This differentiation is correlated with geographical distance, but also with altitude. We also found strong call differentiation among neighbouring family groups. Call differentiation of contact calls was comparatively high in comparison to alarm calls, which showed a lower degree of divergence.

**Conclusion:**

Our data show that call differentiation is governed by geographic isolation as well as altitude. Diverging degrees of call differentiation in contact and alarm calls suggests that both call types are under different selective pressures. Alarm calls are required to be understood by all members of the species across the entire distribution range and thus call differentiation is lower. In contrast, contact calls are more specific and differ even among neighbouring families supporting the maintenance of distinct bird families and groups.

**Electronic supplementary material:**

The online version of this article (10.1186/s12862-018-1222-1) contains supplementary material, which is available to authorized users.

## Background

Species demanding specific habitat characteristics frequently occur in geographically isolated population groups, and thus provide the prerequisite for long-term geographic isolation of populations [1]. In addition, diverging environmental conditions may also drive the isolation of populations [2]. Both drivers may lead to the differentiation of taxa, at molecular, morphological and behavioural level [1, 2]. The interplay of both forces, geographic isolation and diverging environmental conditions, is complemented by the effects of social structures, which may further drive intraspecific differentiation across distribution ranges of taxa [3–5]. Yet, depending on the trait under study, one evolutionary force may be more important than the other.

Traits involved in communication, such as vocalisations are assumed to be under strong selection [3]. For example, bird calls are important behavioural evolutionary traits. It has been shown that call structure is partially heritable, but also culturally transmitted via learning and frequently diverges among local populations, especially when these are geographically or ecologically isolated [4, 5]. Bird vocalisations have an important function in intraspecific communication and may be used to hold contact to other members within a family group [6], or during mating [7]. However, they are also frequently used as alarm signals to warn for predators [8, 9].

In this study we recorded bird calls for the Kenyan endangered endemic Hinde’s Babbler *Turdoides hindei* (Aves, Passeriformes). We considered its entire distribution range across south-eastern Kenya [10] and recorded individuals belonging to 20 families representing the five still existing population groups [10]. The species can be found at humid and cool habitats at higher elevations at the foothills of Mt. Kenya, the Aberdares and the Thika Plateau, but also in the dry and hot lowlands around Machakos, and Kitui with Makueni [11]. Hence, our sampling represents different altitudes and geographically isolated population groups.

*Turdoides hindei* is a cooperative breeder and thus forms distinct family groups with sizes ranging between 2 and 10 individuals [12]. This allows to also study family specific call characteristics, which may play an outstanding role in social species, such as cooperative breeding birds. However, acoustic datasets for such social species across their whole range are rare, but may provide insights into the importance of geographic isolation, environmental conditions (highland versus lowland), and social structures for divergence in acoustic traits.

We observed families and the behaviour of single individuals and recorded contact and alarm calls. In addition, we recorded individuals being members of neighbouring family groups inhabiting the riparian forests of three river systems in the semiarid dry lands in south-east Kenya. We combined the obtained bioacoustics data with characteristics of sites, including geographic isolation, altitude, habitat type, and precipitation. Based on the results we attempt to address the following research questions:

(i) Do call characteristics differ across the distribution range of *T. hindei*?
(ii) Do geographic isolation and/or altitude cause call differentiation?
(iii) Do contact calls and alarm calls vary in their degrees of differentiation?
(iv) Do calls differ among neighbouring families?

## Results

**Fig. 1:**
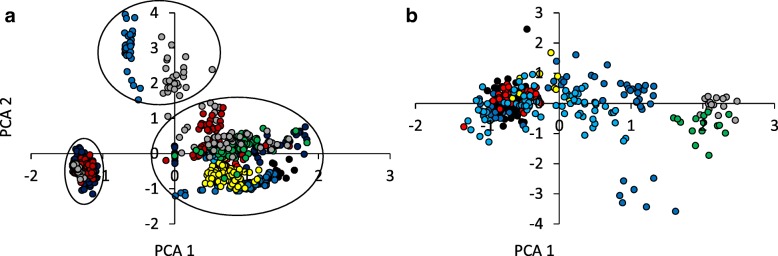
Variance – covariance principal component analyses of (**a**) contact calls and (**b**) alarm calls identified significant (both one-way PERMANOVA: *P* < 0.001) differences of call characteristics among local populations across the species’ entire distribution range (colours are as given in Table 3). Ovals in **a**) denote the three well defined contact call clusters (I to III). PCA1 in **a**) and **b**) was highly correlated with gap length (loading > 0.9). PCA2 in a) correlated with the difference in highest and lowest frequency (loading = 0.72), in **b**) with call length (loading = 0.89); PCA = here Principal Component Axis

Principal component analysis (PCA) grouped the contact (Fig. 1a) and alarm (Fig. 1b) calls into distinct clusters (both one-way PERMANOVA *P* < 0.001). For both, contact and alarm calls, the first PC axis had the highest loading for gap length (loading > 0.9). For contact calls the second axis showed the strongest correlation with differences in highest and lowest frequencies (loading = 0.72); for alarm calls, call length was most important (loading = 0.89). PCA also grouped the local contact calls into three distinct non-overlapping clusters (Fig. 1a). Importantly, these three clusters did not reflect the spatial distribution of sites as cluster I combined individuals from the geographically distant sites Mukrueni and Kitui and cluster II combined individuals from Kitui and Kirinyaga.

However, the family clusters exhibited a highly significant spatial component (one-way PERMANOVA: pseudo-F = 61.2, *P* < 0.001). A Mantel test suggested significant isolation-by-distance for both, contact and alarm calls; geographically close family groups share comparatively similar call characters (contact calls: *r* = 0.13, *P* < 0.001; alarm calls: *r* = 0.16. *P* < 0.001) compared to more distant families. The contact call clusters were neither related to altitude, nor to average precipitation (r^2^ < 0.05, ANOVA P (F_1,12_) > 0.5), but exhibited a moderate, although not significant, latitudinal gradient (contact call: r^2^ = 0.15, ANOVA P(F_1,9_) = 0.21). However, we found a significant difference in call characteristics for contact calls in relation to altitude of the population recorded (highland versus lowland, Table 1); different habitat types (swamp, riparian forest, thicket) (Table 1) led to differences in both, contact and alarm calls. Differences of contact calls among individuals, among family groups and among population clusters are stronger than differences found for alarm calls at all levels. However, we have to note that the geographical distributions of mountain regions (one-way PERMANOVA: pseudo-F = 506, *P* < 0.01) and habitat types (one-way PERMANOVA: pseudo-F = 191, *P* < 0.001) are not random and reflect a longitudinal gradient towards moist lowlands.

**Table 1:** Two-way PERMANOVA detected significant differences in contact and alarm calls between birds at different altitudes (low-, highlands) and living in habitats of different structure (swamp, riparian, thicket)

Factor	Contact calls			Alarm calls		
	d.f.	Pseudo-F	P(F)	d.f.	Pseudo-F	P(F)
Low-/Highland	2	3.34	0.002	2	19.33	< 0.001
Habitat	1	3.89	0.009	1	15.01	< 0.001
Low/High×Habitat	2	1.33	0.73	2	1.36	0.74

Error degrees of freedom (d.f.): contact calls: 495; alarm calls: 560

**Fig. 2:**
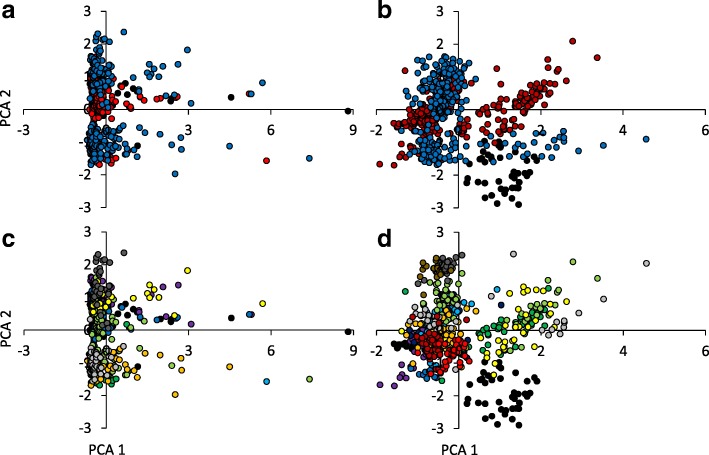
Variance – covariance principal component analyses of contact (**a**, **c**) and alarm (**b**, **d**) calls identified significant (both one-way PERMANOVA: *P* < 0.001) differences of call characteristics from all individuals (irrespective of family membership) recorded along Nzeeu River (*blue* points), Kalundu River (*red*) and Ithani River (*black*) (**a**, **b**), and among single bird families recorded along these rivers (**c**, **d**: family colours are given in Table 3); PCA = here Principal Component Axis

PCA (Fig. 2) and two-way PERMANOVA (Table 2) revealed significant differences (all *P* < 0.001) of contact calls (Fig. 2a, c) and alarm calls (Fig. 2b, d) among individuals of family groups along the three rivers (Nzeeu river, Kalundu river, Ithiani river) (Fig. 2a, b) and also among local family groups (Fig. 2c, d). Two-way PERMANOVA also pointed to highly significant differences in the calling patterns among families along one river (Table 2) and among the three rivers (Table 2).

**Table 2:** Two-way PERMANOVA detected significant differences in contact and alarm calls between birds at different rivers and of different family membership

Factor	Contact calls			Alarm calls		
	d.f.	Pseudo-F	P(F)	d.f.	Pseudo-F	P(F)
River	2	3.54	< 0.001	2	38.16	< 0.001
Family	10	4.34	< 0.001	13	15.48	< 0.001
River×Family	20	0.62	0.10	26	12.36	< 0.001

Error degrees of freedom (d.f.): contact calls: 468; alarm calls: 524

**Fig. 3:**
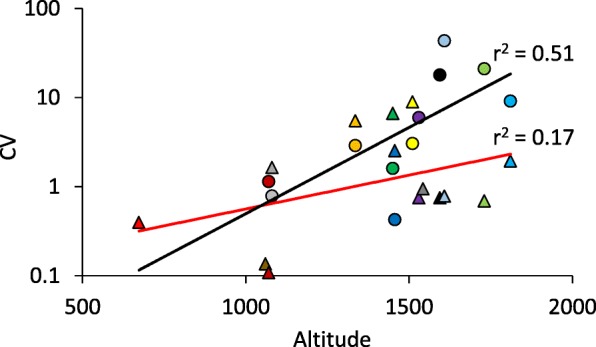
The coefficients of variation (CV) of the first principle component (Fig. 1) increased for contact calls (circles) and alarm calls (triangles) with increasing altitude (in m) of study sites (colours are as given in Table 3). Black exponential regression line (contact calls: permutation *P* = 0.01). Red exponential regression line (alarm calls: permutation *P* = 0.13)

K-means cluster analysis (Additional file 1) resulted in a close accordance of family and cluster membership and confirmed the family specific patterns in contact calls (Additional file 1: Figure S1) and alarm calls (Additional file 1: Figure S2), but also highlighted the variability and overlap among call patterns from local families (Fig. 3, Additional file 1: Figure S1, S2). The intra-family call variability increased with altitude, for both, contact and alarm calls (Fig. 3). Respective Mantel tests based on Euclidean distances in calling pattern and location revealed a significant positive correlation between call dissimilarity and geographical distance (contact calls: *r* = 0.17, permutation *P* < 0.001; alarm calls: *r* = 0.07, permutation *P* < 0.001). In turn, altitude and average precipitation (both *r* < 0.02, *P* > 0.05) did not significantly correlate with call dissimilarity.

## Discussion

We found a significant differentiation of call characteristics of *T. hindei* across the species’ distribution range. Previous studies showed that long-term isolation and subsequent neutral drift effects may drive the evolution of call characteristics of bird calls [13]. Call divergence found for *T. hindei* represents an isolation-by-distance pattern, suggesting an important role of geographic isolation for the evolution of bird call parameters. The evolution of call dialects in geographically isolated bird populations was intensively studied, mainly on bird species living on oceanic islands [14], and on isolated mountain tops [15]. For example, the East African Mountain White-eye *Zosterops poliogaster* occurs exclusively at higher elevations and thus forms mountain specific population groups. This caused long-term isolation with subsequent distinct evolutionary trajectories of bird calls [15]. In contrast, its lowland sibling, the Abyssinian White-eye *Zosterops abyssinicus* is homogenously distributed across interconnected savannahs and thus shows no significant call differentiation [16]. In addition, at localities where both, highland and lowland taxa occur sympatrically, bird call differentiation between both species is strongest. Such character displacement is interpreted as a pre-mating strategy to avoid hybridisation of closely related species when they become sympatric [17]. A study on call evolution of the Cuckoo-dove *Turacoena manadensis* showed significant call divergence across its entire distribution range due to population disjunction in the wake of geological changes accompanied by sea level fluctuations during Pleistocene glacial cycles [18].

In addition to drivers related to geographic isolation, ecological conditions may have strong impact on the evolution of distinct traits [19]. For *T. hindei* we found a significant relationship of call divergence and altitudes. Similar responses on environmental changes in relation to elevation were found in previous studies. For example, song divergence along an altitudinal gradient was detected for the Grey-breasted wood wren *Henicorhina leucophrys* [20]. However, elevation is itself correlated with other habitat parameters, such as humidity and temperature. But, our data do not underline further significant relationships between environmental conditions and call characteristics, or diverging habitat structures. This becomes contrasted by other studies, showing that bird calls differ depending on habitat structures [21]. This is likely at least in part the result of variation in sound propagation in different habitat types [22].

We even detected bird call differentiation between neighbouring bird families of *T. hindei*; differentiation of calls was more pronounced in contact calls than in alarm calls. Bird calls are important to maintain social structures [6, 23–25]. Further, calls are considered to represent an important pre-zygotic mating barrier (e.g. between closely related sibling species, see above) and play a pivotal role in sexual selection. The evolution of call differences within a species might also be of high relevance for species living in social structures, i.e. in family groups of cooperative breeders [26]. Local contact call differentiation may be an important mechanism to recognize the own family group and hence is a crucial cue in species with such social organisation [27]. The relevance of contact calls for the social organisation becomes further supported by our finding of a stronger call differentiation observed in contact calls compared to alarm calls. Alarm calls are considered an altruistic signal which may be under kin selection [28–30].

## Conclusion

Our data suggests that contact calls originally evolved to keep members of a family together [29, 31] and thus often show regional dialects and are assumed to have a strong cultural component [32, 33]. In contrast, alarm calls are more conserved within the species. Thus, our study evidences that both call types are under different selective regimes resulting in different levels of call differentiation.

## Methods

### Study species

Hinde’s Babbler, *Turdoides hindei*, is a bird species endemic to Kenya and is exclusively found in the south-east of the country [11]. The species occurs in the highlands and at lower elevations along rivers and forms disjunct population groups. It is found at the cool and moist Aberdares and the foothills of Mt. Kenya, but also in the dry lowlands around Machakos, Kitui, and Makueni. Some populations occur at intermediate elevations around Thika, Sagana, and Oldonjo Sabuk [10]. *Turdoides hindei* is a cooperative breeder and forms distinct families consisting of two to 10 individuals [12]. The call repertoire includes a contact and an alarm call (JCH, own observations). Typical habitat is dense riparian thicket (including invasive exotic *Lantana camara* shrubs, see Teucher et al. 2015 [34]), in highland and lowland regions [35]. At higher elevations, the species can also be found at coffee plantations (JCH, own observations). The distribution of suitable habitat creates a long-term disjunction of this species. Agricultural intensification and clearance of shrubs along rivers with subsequent loss of habitats and the fragmentation of the remaining thicket has caused a severe decline of *T. hindei* populations within population groups, mainly across the semiarid lowland populations around Kitui [10]. Due to this negative population trend, *T. hindei* is classified as globally vulnerable according the IUCN Red List [36, 37].

### Study region

**Fig. 4:**
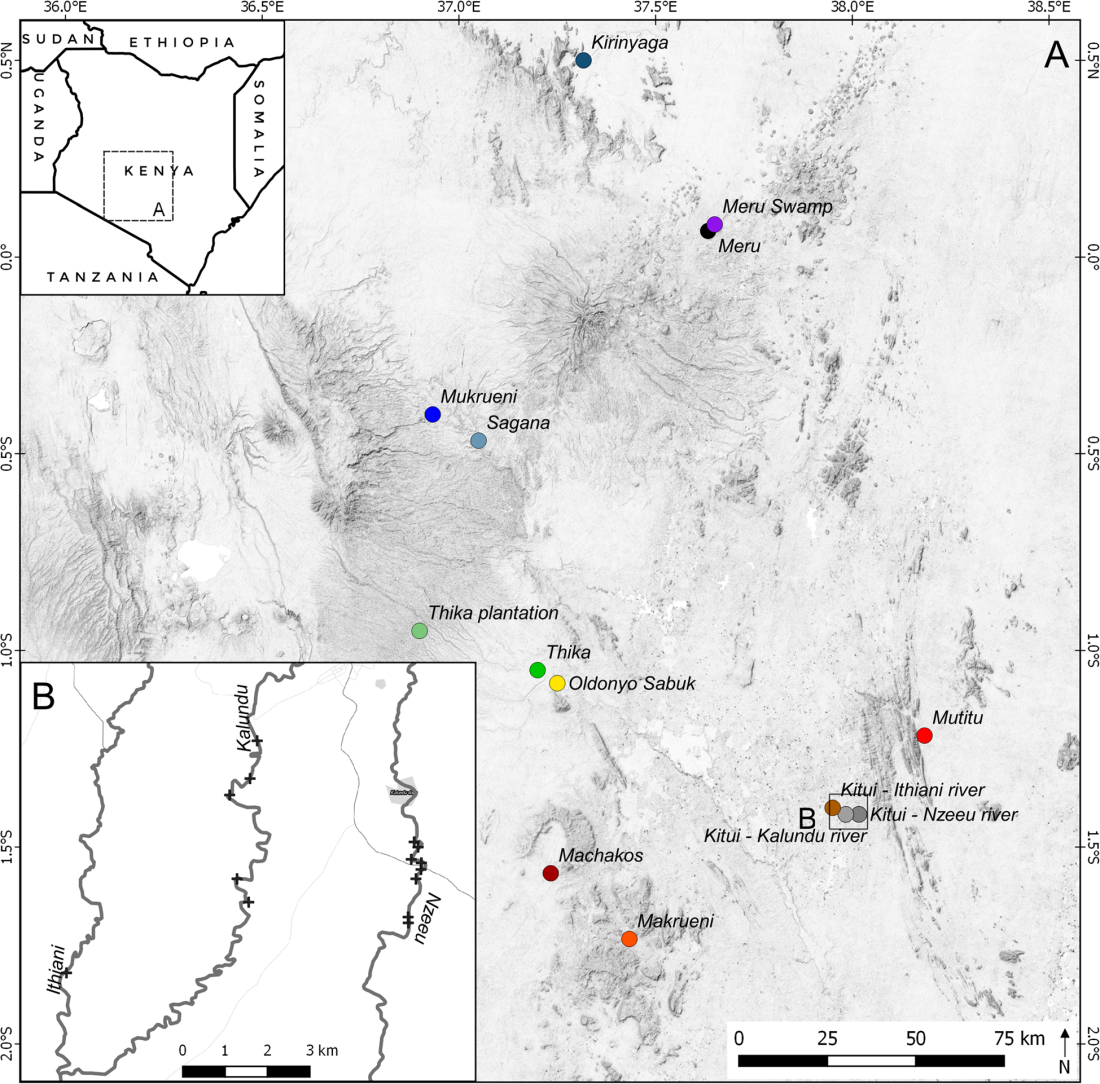
Study region in Kenya (small inlet map of Kenya, shaded in grey), and geographic locations where bird calls of *Turdoides hindei* were recorded, including a detailed map showing the three rivers Nzeeu, Kalundu, Ithiani, along which families were observed and bird calls were recorded. Names and colors are congruent with other figures and tables

Our study region covers the entire distribution range (south-eastern Kenya) with populations found along rivers, but also in agricultural land. Highland populations are represented by the following sites: Kirinyaga, Sagana, Meru, Meru swamp, Mukrueni, Thika, Thika plantation, Oldonyo Sabuk. These sites are characterised by comparatively cool and wet climate. Populations from the hot dryland region are Mutitu, Machakos, Makrueni, Kitui (including the following three rivers: Nzeeu, Kalundu, and Ithiani River). Further details are given in Table 3. Sites are displayed in Fig. 4.

**Table 3:** Overview of all populations of *Turdoides hindei* for which bird calls were recorded. Given is the locality name, colour code (coinciding with Figures), GPS coordinates, altitude, habitat type, average precipitation, estimated number of members of the respective family recorded, number of contact calls and alarm calls analysed

Locality	Colour-code	GPS-Coordinates	Altitude (m)	Habitat type	Average precipitation	*N* family members	*N* contact calls	*N* alarm calls
Kirinyaga	Dark blue	0°30’N; 37°19′E	1456	Thicket	450	10	182	20
Sagana	Light blue	0°28’S; 37°03′E	1608	Riparian	950	> 5	13	76
Meru	Black	0°04’N; 37°38′E	1594	Swamp	1500	< 5	16	60
Meru swamp	Violet	0°05’N; 37°39′E	1530	Swamp	1300	< 5	42	32
Mukrueni	Blue	0°24’S; 36°56′E	1810	Thicket	950	> 10	94	49
Thika	Green	1°03’S; 37°12′E	1450	Thicket	850	< 5	81	15
Thika plantation	Grass green	0°57’S; 36°54′E	1730	Riparian	1500	> 5	84	62
Oldonyo Sabuk	Yellow	1°05’S; 37°15′E	1510	Riparian	850	> 5	112	9
Mutitu	Red	1°13’S; 38°11′E	673	Riparian	750	< 5	20	34
Machakos	Dark red	1°34’S; 37°14′E	1542	Riparian	650	< 5	11	28
Makrueni	Orange	1°44’S; 37°26′E	1335	Thicket	850	< 5	37	42
Kitui – Nzeeu river	Dark grey	1°25’S; 38°01′E	1070	Riparian	1100	> 5	233	270
Kitui – Kalundu river	Light grey	1°25’S; 37°59′E	1080	Riparian	950	> 5	225	254
Kitui – Ithiani river	Brown	1°24’S; 37°57′E	1060	Riparian	950	< 5	43	42

### Data collection

Bird calls were recorded in 2013 and 2014 year round (including the dry and rainy season), between 6 am and 6 pm with a NTG2 Røde (Australia) dual powered directional condenser microphone. The input level was operated manually and adjusted to 100%. A digital Zoom-H4 recorder was used to save the calls as stereo *wav*-files. Calls of the birds were recorded with approximately 10 m distance between the microphone and the target individual or family group. While recording we observed the members of a family group and assigned calls to individuals, as far as possible. Furthermore, we classified bird calls into two call types, according the activity of the bird observed, and the sound of the call: contact calls, being emitted from members while feeding and foraging to keep contact with members of the same family; alarm calls, being emitted when an individual got lost and isolated from the family group. Both contact and alarm calls are mostly emitted in series and regular intervals. We recorded bird calls of 20 families.

**Fig. 5:**
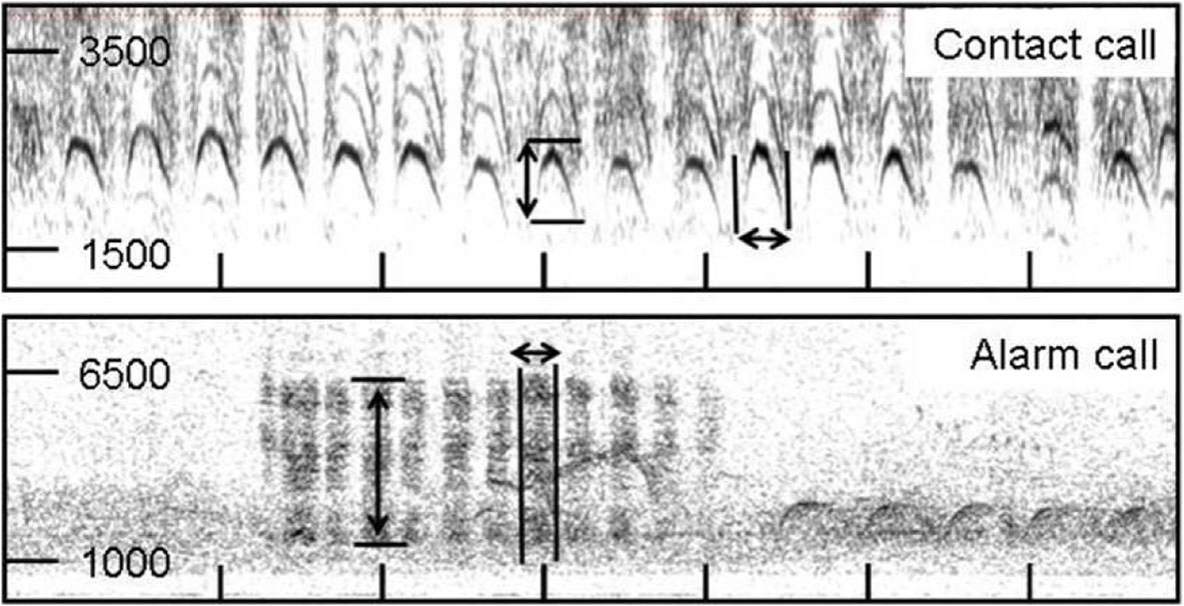
Typical sonogram of an alarm call (above) and a contact call (below) of *Turdoides hindei*, recorded south of Kitui. The following call characteristics were analysed: highest and lowest frequency (circles), and frequency range and length of single calls (arrows)

Recordings of high quality were processed with the programme AUDACITY v. 2.01 (Audacity Development Team). Calls affected by strong background noise or overlapping with other calls from the same (or other) species were excluded from further analyses. After deleting calls of bad quality, a total number of 2177 calls remained (at least 9 records of each call type per family emitted from different individuals, except of two families without contact call recording). We analysed the following call characteristics: lowest frequency, highest frequency, frequency range (distance between lowest and highest frequency), and the length of each call. We neither took into consideration the total number of bouts emitted, nor the total length of series of bouts or the duration of single gaps between bouts, as these parameters are strongly influenced by the level of excitement of individuals (JCH, observations), and thus the temporary state of individuals of a family may overlay general call characteristics. Spectral analyses were performed blind to site (i.e. population) using the programme PRAAT v. 5.2.15 [38]. Range of frequency (Hz) and the dynamic range (dB) were adjusted depending on the background noise in the spectrogram settings. Typical sonograms of contact calls and alarm calls are presented in Fig. 5.

### Statistics

Due to the pseudo-replicated data structure (non-independence among calls within each family and possible spatial autocorrelation of data) we used permutation tests based on one- and two-way PERMANOVA [39] and Mantel correlation (Euclidean distances) to link individual bird vocalisations (lowest and highest frequency, frequency range and call length) for both call types (contact call and alarm call) to geographic distances among populations (isolation by distance), family membership, average precipitation (data on precipitation from local weather stations), habitat type (swamp, riparian forest, thicket) and altitude. We note that family membership, and climatic and habitat variables were not spatially autocorrelated at the 5% error level. Consequently, we did not include spatial distance into the PERMANOVA and Mantel analyses.

Variance-covariance based principal components analysis (PCA) served to discriminate bird vocalisations among population clusters and local families. Among and within family variability in bird call patterns were studied using k-means clustering. K-means grouped all individuals according to their contact and alarm calls into 14 predefined clusters, the number of observed families. If families have distinct calling structures a close accordance between cluster and family membership should result. Calculations were done with Primer 7 [40] and Statistica 12 (Statsoft, Tolsa, OK, USA).

## Additional file

Additional file 1: Numbers of classification within each family of a k-means clustering of contact calls. Colors coincide with the colors given in the main text. Raw data (wav-files) of bird calls recorded and analyzed are available at figshare.com, https://figshare.com/s/ab27b9a4c1aca6897825. (DOCX 144 kb)
