# Supplementary material for: Evolution of contact and alarm calls in the Kenyan endemic Hinde’s babbler (Aves: Passeriformes)
Source: BMC Evol Biol. 2018 Jul 17;18:112. doi: 10.1186/s12862-018-1222-1 (PMC6050726; doi:10.1186/s12862-018-1222-1)
Supplement: Supplementary file 1 — Numbers of classification within each family of a k-means clustering of contact calls. Colors coincide with the colors given in the main text. Raw data (wav-files) of bird calls recorded and analyzed are available at figshare.com, https://figshare.com/s/ab27b9a4c1aca6897825. (DOCX 144 kb) [file 12862_2018_1222_MOESM1_ESM.docx]

Additional file 1

**Evolution of contact and alarm calls in the Kenyan endemic Hinde´s Babbler
(Aves: Passeriformes)**

Jan Christian Habel Martin Husemann, Werner Ulrich

Figure S1: Numbers of classification within each family of a k-means clustering of contact calls. Colours coincide with the colours given in the main text.

Figure S2: Numbers of classification within each family of a k-means clustering of alarm calls. Colours coincide with the colours in the main text.
